# Intravenous Infusion of Lidocaine for Bowel Function Recovery After Major Colorectal Surgery: A Critical Appraisal Through Updated Meta-Analysis, Trial Sequential Analysis, Certainty of Evidence, and Meta-Regression

**DOI:** 10.3389/fmed.2021.759215

**Published:** 2022-01-27

**Authors:** Po-Chuan Chen, Chao-Han Lai, Ching-Ju Fang, Pei Chun Lai, Yen Ta Huang

**Affiliations:** ^1^Department of Surgery, National Cheng Kung University Hospital, College of Medicine, National Cheng Kung University, Tainan, Taiwan; ^2^Department of Biochemistry and Molecular Biology, College of Medicine, National Cheng Kung University, Tainan, Taiwan; ^3^Department of Biostatistics, Vanderbilt University Medical Center, Nashville, TN, United States; ^4^Medical Library, National Cheng Kung University, Tainan, Taiwan; ^5^Department of Secretariat, National Cheng Kung University Hospital, College of Medicine, National Cheng Kung University, Tainan, Taiwan; ^6^Education Center, National Cheng Kung University Hospital, College of Medicine, National Cheng Kung University, Tainan, Taiwan

**Keywords:** lidocaine, colorectal surgery, flatus, defecation, meta-analysis, trial sequential analysis, meta-regression

## Abstract

**Background:**

Intravenous infusion of lidocaine (IVF-Lido) during the perioperative period is an option to accelerate bowel function recovery after major colorectal surgery. However, previous meta-analyses have shown inconsistent conclusions. Recent randomized controlled trials (RCTs) have been reported after the publication of a previous meta-analysis.

**Aim:**

We conducted an updated and comprehensive meta-analysis to determine the effects of IVF-Lido on time to first flatus and defecation after major colorectal surgery.

**Methods:**

We performed a systematic review according to the Preferred Reporting Items for the Systematic Reviews and Meta-Analysis Protocols 2020 guideline. Only RCTs were included. The revised Cochrane risk-of-bias tool was chosen for appraisal. Meta-analysis with meta-regression and trial sequential analysis was carried out. The Doi plot was presented to evaluate publication bias. The Grading of Recommendations, Assessment, Development, and Evaluations (GRADE) methodology was executed to evaluate the certainty of evidence (CoE).

**Results:**

Thirteen RCTs with 696 participants were enrolled. IVF-Lido significantly decreased the time to first flatus [mean difference (MD) = −6.03 h; 95% confidence interval (CI): (−8.80, −3.26)] and first defecation [MD = −10.49 h; 95% CI: (−15.58, −5.41)]. Trial sequential analysis yielded identical results and ampleness of required information sizes. No obviousness in publication bias was detected, and the CoE in GRADE was low in both outcomes. Meta-regression showed that a significantly shorter time to the first defecation was associated with studies with more improvement in pain control in comparison of two groups and better-improved analgesia in the control group.

**Conclusions:**

We discretionarily suggest the use of IVF-Lido on postoperative bowel function recovery following major colorectal surgery. Beyond the analgesic effects, IVF-Lido might have additional benefits when postoperative pain relief has already been achieved. Considering the high heterogeneity in this updated meta-analysis, more RCTs are needed.

**Systematic Review Registration:**

https://inplasy.com/inplasy-2020-7-0023/, INPLASY [202070023].

## Introduction

With the current popularity of enhanced recovery after surgery (ERAS) in the colorectal surgery field, many aspects of perioperative interventions have been proposed to accelerate patients' bowel function recovery (1). Intravenous infusion of lidocaine (IVF-Lido) during the perioperative period is among the most promising options being investigated (1–7). After major colorectal surgery, enhanced bowel function recovery was observed by IVF-Lido in some trials (8–10). However, in other literature, IVF-Lido did not influence bowel function recovery (11–16). From a physician's point of view, these conflicting findings are intriguing.

When randomized controlled trials involving a broad spectrum of intra-abdominal procedures and other surgeries were selected for meta-analysis, uncertain results on the beneficial effects of IVF-Lido on postoperative pain control or bowel function recovery were obtained in the recent Cochrane Database of Systematic Reviews (5). When we focused only on the effect of IVF-Lido for bowel function recovery in major colorectal surgery, three recently published meta-analyses were found (17–19). However, two common limitations were observed in these reports. Firstly, the influence of risk of bias (RoB) in enrolled RCTs, which is one of the critical domains in the appraisal tool “A Measurement Tool to Assess Systematic Reviews 2 (AMSTAR2),” was not considered (20). Second, no grading was performed on the certainty of evidence (CoE) for their results. These limitations could all easily skew the result directions if not handled properly. Besides, several new RCTs conducted specifically for this topic had not been previously enrolled in meta-analyses (16, 21–23), indicating the need for an updated meta-analysis. For a more comprehensive appraisal, we performed trial sequential analysis (TSA) (24) and meta-regression for thorough analyses that will result in precise evidence. TSA, an increasing application of statical methodology in meta-analysis, can provide more cautious results by repetitive and cumulative testing (25). Additionally, we provided the CoE of each study endpoint using Grading of Recommendations, Assessment, Development and Evaluation (GRADE) methodology (26).

## Materials and Methods

### Protocol and Registration

This systematic review followed the Preferred Reporting Items for Systematic Reviews and Meta-Analysis Protocols (PRISMA) 2020 guideline and the Cochrane Handbook (27–29) and further accessed the quality based on AMSTAR2 (20). We registered our protocol on a website for protocol registration of systematic review INPLASY with the registration number of INPLASY 202070023 (doi: 10.37766/inplasy2020.7.0023). The protocol was updated, and changes were recorded until 25 July 2021.

The structured search strategy was developed by a librarian author (Fang CJ), and results were retrieved from seven databases, namely, Embase, MEDLINE, Cochrane CENTRAL, Scopus, China National Knowledge Infrastructure, Index to Taiwan Periodical Literature System, and WHO International Clinical Trials Registry Platform (ICTRP) with no language restrictions until 25 July 2021. Reference lists of relevant articles and conference abstracts were reviewed to identify additional studies.

The two key concepts “colorectal surgery” and “lidocaine,” along with their synonyms (42 free-text terms plus truncation symbols when appropriate) and controlled vocabulary (19 MeSH terms and 22 Emtree terms), were used. Supplementary Table 1 shows the comprehensive search strategy. Another author (Chen PC) also joined the search.

### Study Selection and Exclusion

Two independent authors (Chen PC and Fang CJ) examined the titles and abstracts of these studies. Then, the full texts of relevant studies were retrieved. The inclusion criteria were as follows: (1) RCTs that involved patients undergoing major colorectal surgery, (2) intervention and control groups treated with IVF-Lido and IVF-saline, respectively, and (3) the primary outcome consists of the bowel function recovery under IVF-Lido, including the time to first flatus passage and the time to the first defecation after surgery. Non-RCTs were not enrolled to avoid confounding and selection bias.

### Data Collection and Quality Assessment

Data were extracted from eligible studies included by two authors (Chen PC and Fang CJ). The extracted data included first author, publication year, country of the study, source of funding number of patients, procedure type, IVF-Lido dosage in intervention groups, drugs for maintenance of general anesthesia, additional uses of perioperative analgesics, outcomes of bowel function recovery, and visual pain analog scores (VAS) or numerical rating scales (NRS). The RoB was independently assessed by two authors (Lai PC and Huang YT) by using the RoB tool 2.0 for RCTs (30). Divergences were resolved by consensus.

### Data Synthesis and Statistical Analysis

The meta-analysis was performed using Review Manager (RevMan) version 5.3 (Copenhagen: The Nordic Cochrane Center, The Cochrane Collaboration, 2014). Continuous variables were pooled as mean difference (MD) with 95% confidence intervals (CIs). A random-effects model was chosen for the meta-analysis. Subgroup analyses were further performed based on the overall RoB of enrolled RCTs based on the requirement of AMSTAR2. Heterogeneities among studies were evaluated using the I-square (I^2^) statistics. *A priori* power of the meta-analysis was calculated by the R package “dmetar” with the random-effects model (30).

When a meta-analysis using sparse data, type I errors may occur due to low methodological quality, outcome measure bias, publication bias, small trial bias, or random errors. The use of TSA provides more information on the precision and uncertainty of meta-analysis results (25). TSA was conducted using TSA version 0.9.5.10 beta (Copenhagen Trial Unit, Center for Clinical Intervention Research, Rigshospitalet, Copenhagen, Denmark). O'Brien–Fleming α-spending monitoring boundaries were applied for hypothesis testing. Types I and II errors were set at 5 and 20%, respectively, with the random-effects model by using the DerSimonian and Laird method. The calculated required information size considered the choice of the empirical item for MD, and model-based variance item was chosen for heterogeneity calculation as diversity (D^2^). The TSA result was presented as MD and α-spending-adjusted CI.

Sensitivity analysis is a methodology to deal with ranges of values for decisions that were unclear (28). Sensitivity analysis with meta-regression was conducted using the R package “metafor”-based software OpenMetaAnalyst with the random-effects model (31). Covariates for meta-regression included the types of surgery, differences (Δ) of visual analog scale or numeric rating scale (VAS/NRS) between two groups 24 h after surgery at rest and baseline analgesic status (control group). Publication bias was assessed through visual inspection by using funnel plots and quantified using the Doi plot with Luis Furuya–Kanamori (LFK) index (MetaXL version 5.3, EpiGear International Pvt., Ltd.) (32). The values of LFK index outside the interval between −1 and +1 were as associated with asymmetry, indicating the probability of publication bias.

### Grading of the Certainty of Evidence

Not only to yield the statistical results but also to state the level of evidence becomes an essential presentation in the research of evidence-based medicine (26). We assessed the outcomes by using GRADE methodology (26). The overall CoE was evaluated using five downgrading domains. The level of evidence was classified as high, moderate, low, or very low. Grading was performed using GRADEpro software (available from http://www.gradepro.org).

## Results

**Figure 1:**
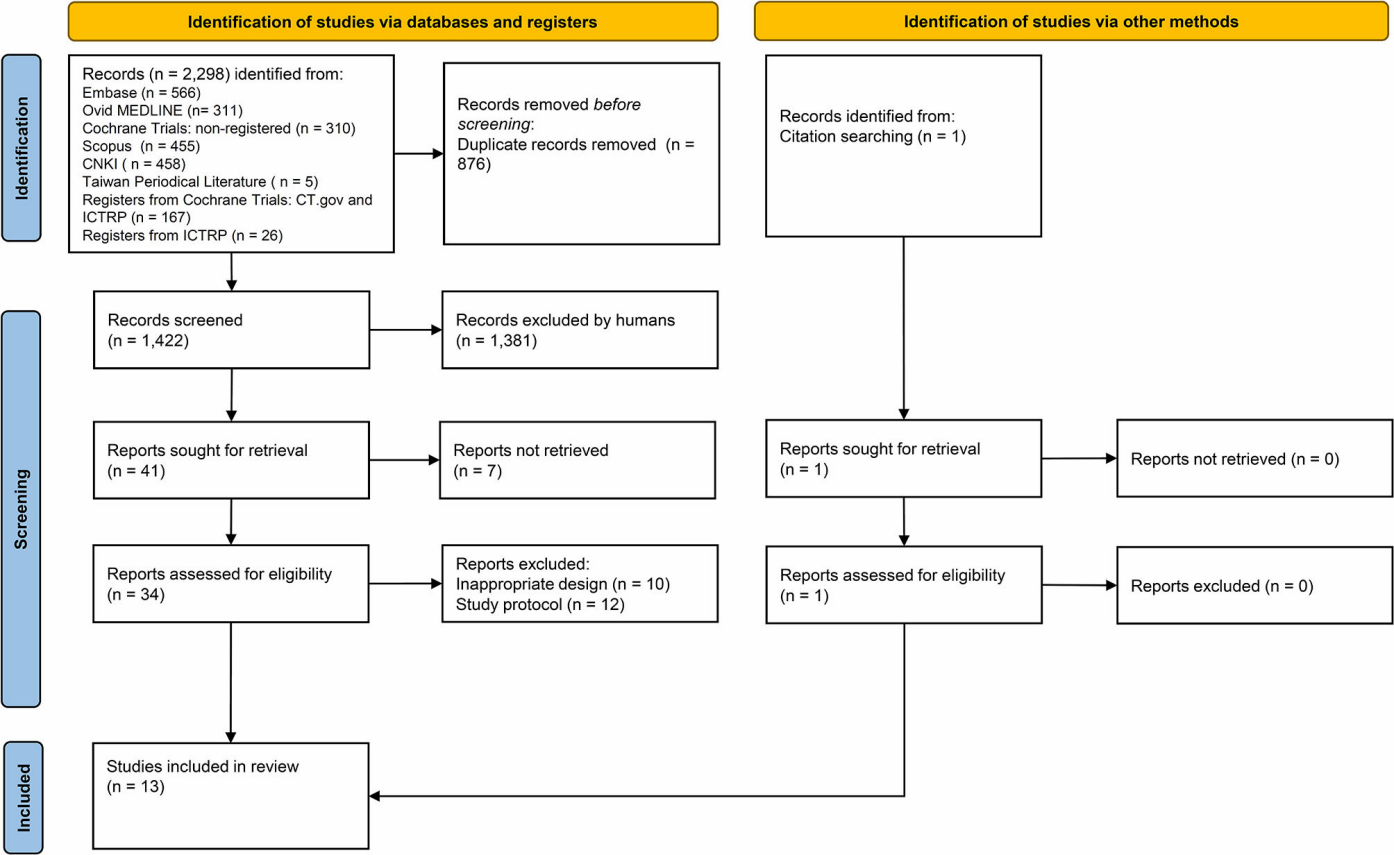
A PRISMA 2020 flow chart of the study selection process.

The initial literature search identified 2,298 potentially eligible articles, among which 13 studies were finally enrolled in our meta-analysis (Supplementary Table 1, Figure 1). These 13 RCTs were published between 2006 and 2020. The types of operative approaches included open surgery in five studies, laparoscopic surgery in seven studies, and robotic surgery in one study. High diversity was noted among studies on the use of perioperative medications, including the loading dosage of IVF-Lido, total dosage and duration of perioperative IVF-Lido, drugs for maintenance of anesthesia, and additional drugs for pain management (Table 1).

**Table 1:** Details of RCTs included in our meta-analysis.

**References**	**Country**	**Sources of funding**	**Number of participants**	**Procedure type**	**Dosage of lidocaine**	**Additional drugs for pain control**	**Drugs for maintenance of general analgesia**	**First flatus hours, mean (SD) or median (IQR)**	**First defecation hours, mean (SD) or median (IQR)**	**VAS/NRS score (24 h at rest)**
Kuo et al. (8)	Taiwan	Academic	IVF-Lido: 20	Open surgery	Loading 2 mg/kg, then 3 mg/kg/h for 72 h	Morphine and ropivacaine PCEA	Desflurane, fentanyl	60.2 (5.8)	N/I	2.4
			IVF-saline: 20					71.7 (4.7)	N/I	2.9
Kaba et al. (9)	Belgium	Academic	IVF-Lido: 20	Laparoscopic surgery	Loading 1.5 mg/kg IV, then 2 mg/kg/h intra-op, then 1.33 mg mg/kg/h for 24 h	Propacetamol IV, ketorolac IV, piritramide PCA	Sevoflurane, sufentanil	17 (11–24)	28 (24–37)	0.7
			IVF-saline: 20					28 (25–33)	51 (41–70)	1.7
Herroeder et al. (11)	Germany	Academic	IVF-Lido: 31	Open surgery	Loading 1.5 mg/kg, then 2 mg/min for 4 h	Piritramide PCA, metamizole, paracetamol	Sevoflurane, nitrous oxide	50 (20)	66.6 (26.4)	3
			IVF-saline: 29					60 (27)	82.1 (33.8)	3
Zou et al. (21)	China	N/I	IVF-Lido: 30	Open surgery	Loading 1.5 mg/kg, then 1.5 mg/kg/h for 12 h	Morphine IV	Sevoflurane, sufentanil, vecuronium bromide	23 (6)	31 (7)	2.1
			IVF-saline: 30					28 (7)	43 (9)	2.2
Elhafz et al. (33)	Egypt	N/I	IVF-Lido: 9	Laparoscopic surgery	2 mg/min if BW>70 kg or 1 mg/min if BW <70 Kg, till bowel function recovery	Morphine PCA	Isoflurane	39.6 (12.7)	61.4 (9.5)	2.7
			IVF-saline: 9					51.4 (14.2)	82.3 (10.1)	4.1
Tikuisis et al. (10)	Lithuania	Academic	IVF-Lido: 30	Laparoscopic surgery	Loading 1.5 mg/kg, then 2 mg/kg/h intra-op, then 1 mg mg/kg/h for 24 h	Fentanyl IVF	Sevoflurane, fentanyl	26.97 (2.3)	N/I	2.6
			IVF-saline: 30					32.93 (2.86)	N/I	4
Kim et al. (12)	South Korea	Academic	IVF-Lido: 32	Laparoscopic surgery	Loading 1 mg/kg, then 1 mg/kg/h for 24 h	Ketorolac IVF	Sevoflurane, nitrous oxide	58 (48–72.8)	78 (59.3–93.8)	5.7
			IVF-saline: 36					48 (36–73.5)	73.5 (47–106.5)	5.7
Staikou et al. (13)	Greece	Academic	IVF-Lido: 20	Open surgery	Loading 1.5 mg/kg, then 2 mg/kg/h intra-op	Morphine and ropivacaine PCA, paracetamol, lornoxicam	Desflurane, remifentail	72.4 (6.54)	N/I	1
			IVF-saline: 20					73.6 (21)	N/I	1
Dewinter et al. (14)	Belgium	Academic	IVF-Lido: 50	Laparoscopic surgery	Loading 1.5 mg/kg, then 1.5 mg/kg/h for 4 h	Acetaminophen IV, ketorolac IV, morphine IV and PCIA	Sevoflurane	24 (24–48)	72 (48–120)	4.7
			IVF-saline: 25					24 (24–48)	72 (48–120)	4
Ho et al. (15)	Australia	Academic	IVF-Lido: 28	Open surgery	Loading 1.5 mg/kg, then 1 mg/kg/h for 48 h	Fentanyl IVF and PCIA, NSAID, ketamine	Sevoflurane, desflurane	67.7 (38.5)	80.1 (42.2)	3
			IVF-saline: 29					70 (31.2)	82.5 (40.4)	4
Zhao et al. (23)	China	Academic	IVF-Lido: 20	Laparoscopic surgery	Loading 2 mg/kg, then 1.5 mg/kg/h intra-op	Flurbiprofen IV, Pethidine IM	Sevoflurane, propofol	15.7 (1.53)	27.5 (5.72)	N/I
			IVF-saline: 20					25.35 (3.4)	32.1 (6.46)	N/I
Wang (22)	China	N/I	IVF-Lido: 40	Laparoscopic surgery	Loading 1 mg/kg, then 1 mg/kg/h intra-op	Sufentanil IVF	Propofol, rocuronium, sufentanil	18.2 (5.5)	32.7 (6.4)	N/I
			IVF-saline: 40					28.2 (6.4)	45.2 (9.3)	N/I
Herzog et al. (16)	Denmark	Academic	IVF-Lido: 29	Robotic surgery	Loading 1.5 mg/kg, then 1,500 mg/h till PACU for 2 h	Morphine IV, Paracetamol, NSAID	Sufentanil, desflurane	32 (24–40)	N/I	2
			IVF-saline: 29					34 (26–48)	N/I	2

*Lido, lidocaine; SD, standard deviation; IQR, interquartile range; VAS, visual analogue scale; NRS, numeric rating scale; h, hour; N/I, no information; PACU, post-anesthesia care unit; op, operation; IV(F), intravenous (infusion); IM, intramuscular; PC(E or I)A, patient controlled (epidural or intravenous) anesthesia*.

### RoB Assessment

**Figure 2:**
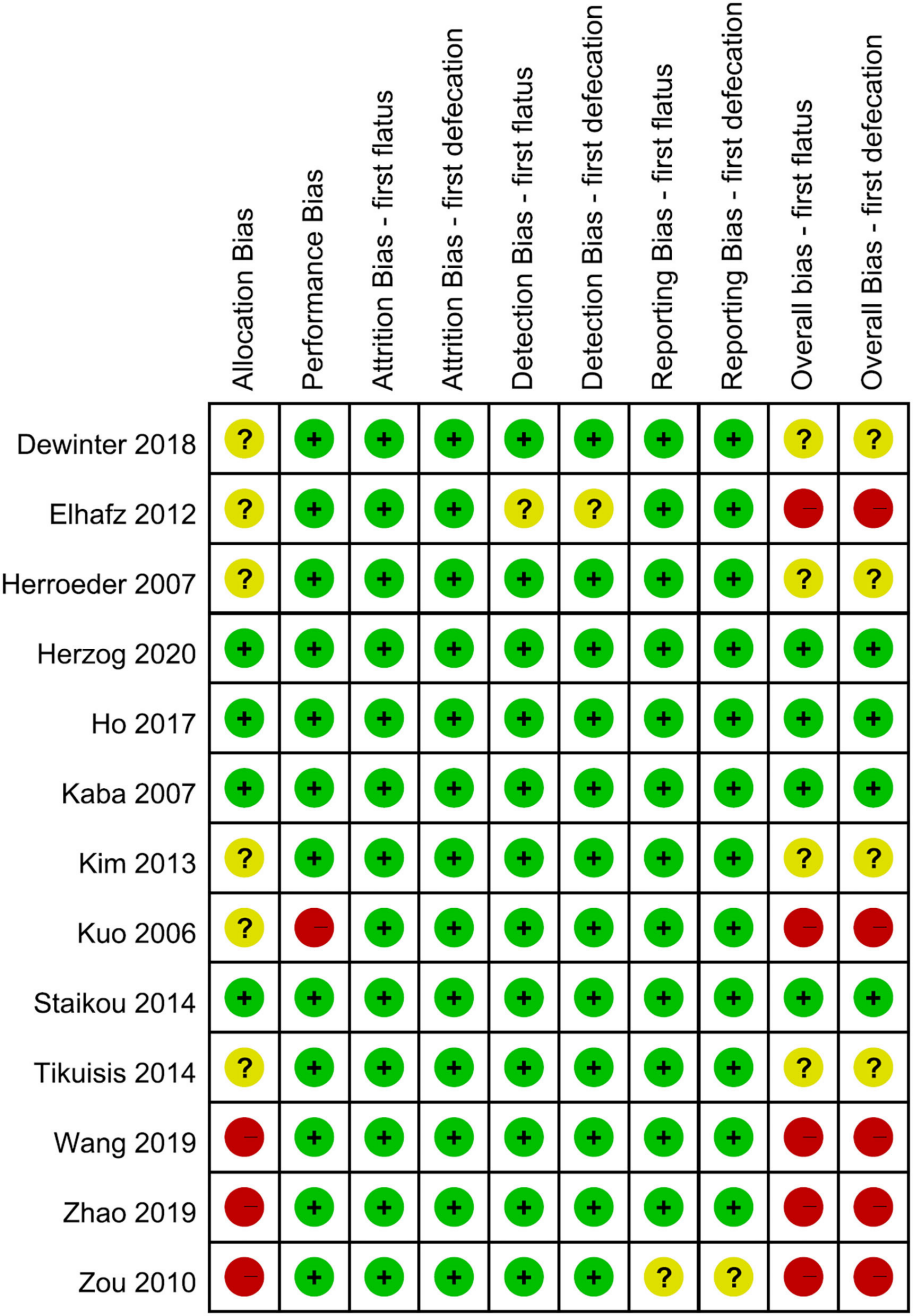
Risk of bias across all included RCTs using the Cochrane RoB 2.0 tool.

Each domain and the overall RoB of included RCTs are shown in Figure 2. Eight RCTs were rated as low (9, 13, 15, 16) or some-concern overall RoB (10–12, 14). Four RCTs (8, 21–23) were rated as high overall RoB based on the high RoB in one domain, such as allocation or performance bias. Based on the expounding of RoB 2.0, the study by Elhafz et al. (33) was rated as a high overall RoB because of some-concerned RoB in the domains of both allocation and detection bias. Six studies have some concerns in allocation bias, because no information is available about concealment (8, 10–12, 14, 33), and the three other studies were classified as high RoB because of the lack of information about both concealment and randomization (21–23). Regarding the domain of performance bias, only one study was rated as high RoB, because no information is available about the participants and personnel being blinded, and the authors did not appropriately analyze the effect of adherence (8). In the domain of performance bias, Elhafz et al. (33) did not mention the awareness of the outcome assessor, leading to some-concerned RoB. For the domain of reporting bias, we rated the study from Zou et al. (21) as some-concerned RoB, because no pre-specified plan was reported. In summary, five out of thirteen RCTs were rated as high overall RoB (8, 21–23, 33), and the influence of pooled estimates from not low portion of high-overall-RoB studies should be considered.

### Pooled Effects of IVF-Lido on Bowel Function Recovery

**Figure 3:**
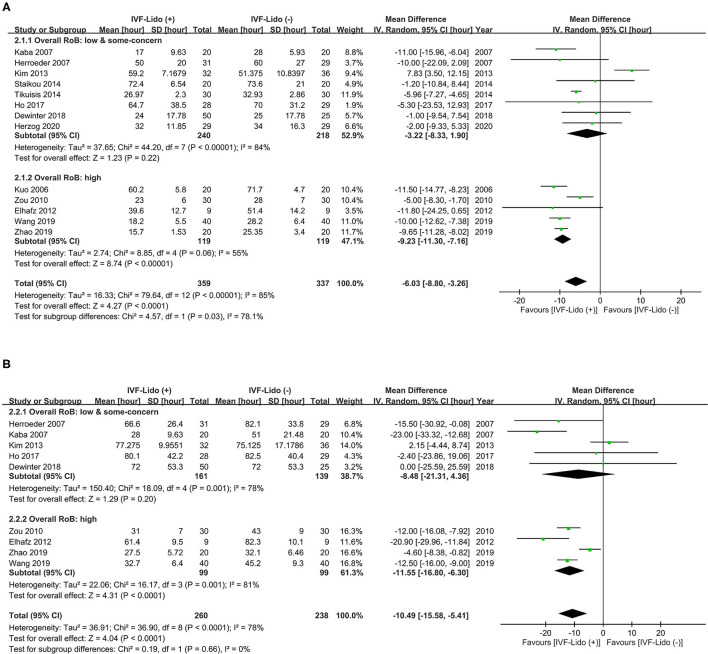
Effect of intravenous infusion of lidocaine on the time (hours) to **(A)** first flatus and **(B)** defecation. A Forest plot with subgroup analysis is divided into two groups, namely, high and low/some-concerned overall RoB. IVF-Lido, intravenous infusion of lidocaine; RoB, risk of bias; SD, standard deviation; CI, confidence interval.

Pooled effects of IVF-Lido from all the enrolled RCTs yielded significant shortening in time to first flatus [MD = −6.03 h, 95% CI: (−8.80, −3.26), I^2^ = 85%, Figure 3A] and time to first defecation [MD = −10.49 h, 95% CI: (−15.58, −5.41), I^2^ = 78%, Figure 3B]. Considering that high heterogeneity was observed in both outcomes, we further performed subgroup analysis based upon different RoBs and population of enrolled studies.

Significant shortening in time to first flatus by IVF-Lido was observed in the pooled effects of RCTs with high overall RoB [MD = −9.23 h, 95% CI: (−11.30, −7.16), I^2^ = 55%, Figure 3A]. By contrast, the pooled effect of IVF-Lido on time to first flatus became non-significant when only eight RCTs with low/some-concerned overall RoB were enrolled [MD = −3.22 h, 95% CI: (−8.33, 1.90), I^2^ = 84%, Figure 3A]. A test for subgroup differences revealed high heterogeneity (I^2^ = 78.1%, *p* = 0.03), indicating discordant results between RCTs with low/some-concerned and high overall RoB (Figure 3A). The pooled results between RCTs with high and low/some-concerned overall RoB showed a statistically significant difference in time to first flatus (*p* = 0.031, Supplementary Figure 1) by using meta-regression analysis, but such difference was underpowered (0.708). In summary, significant shortening in time to first flatus by IVF-Lido from a total of 13 RCTs might be contributed from 8 high-overall-RoB RCTs. However, we cannot perform subsequent analyses by the enrolment of RCTs with low/some-concerned overall RoB exclusively because the difference between RCTs with high and low/some-concerned overall RoB was not conclusive.

Similarly, the pooled effects of IVF-Lido on time to the first defecation [MD = −8.48 h, 95% CI: (−21.31, 4.36) I^2^ = 78%, Figure 3B] were non-significant based on five RCTs with low/some concerned overall RoB, when RCTs with high overall RoB were excluded. However, this subgroup pooled estimate was underpowered (0.540). A test for subgroup differences revealed low heterogeneity (I^2^ = 0%, *P* = 0.66) between RCTs with low/some-concerned and high overall RoB (Figure 3B). When all RCTs were enrolled, the power was 1.00. Again, insufficient cases and inconclusive results may occur when only enrolment of 5 RCTs with low/some-concerned overall RoB. Therefore, we performed subsequent analyses with the enrolment of all included studies in the endpoint of time to the first defecation.

We also divided the included studies into Asian and non-Asian groups because near half (six in thirteen) of the enrolled studies were conducted in Asia. Significant shortening in time to first flatus (Supplementary Figure 2A) and defecation (Supplementary Figure 2B) in both subgroups was depicted. Low heterogeneity (I^2^ = 30 and 1% in time to first flatus and defecation, respectively) was presented in both endpoints of the non-Asian group. In contrast, high heterogeneity (I^2^ = 92 and 82% in time to first flatus and defecation, respectively) was observed in both endpoints of the Asian group. The pooled estimates in time to first flatus were similar between two groups (−5.88 vs. −6.18 h in the Asian and non-Asian groups, respectively), but the pooled estimates in time to the first defecation were much shorter in the non-Asian group (−6.93 vs. −19.61 h in the Asian and non-Asian groups, respectively). It is worth noting that four out of six Asian studies were rated as high overall RoB.

### Pooled Effect of IVF-Lido on Bowel Function Recovery in TSA

**Figure 4:**
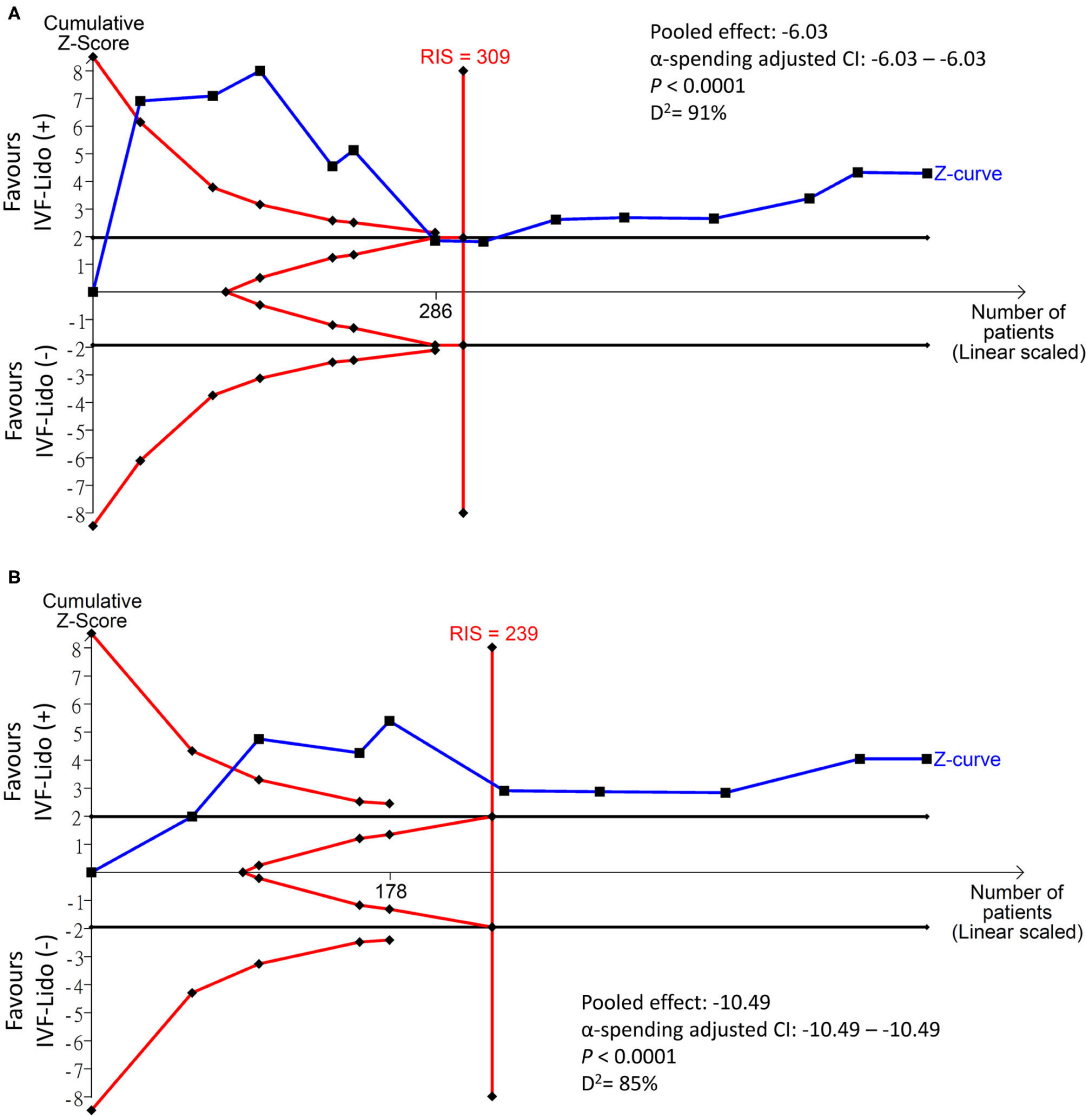
Trial sequential analysis in the time (hours) to **(A)** first flatus and **(B)** defecation. IVF-Lido, intravenous infusion of lidocaine; RIS, required information size.

The cumulative effect of TSA was considered true positive if the Z curve crossed the O'Brien–Fleming monitoring boundaries and was considered true negative if the Z curve entered the futility area. The Z curves in the study endpoints of time to first flatus (Figure 4A) and time to first defecation (Figure 4B) straddled the O'Brien–Fleming α-spending monitoring boundaries since the first and second studies, respectively. Interestingly, the Z curve of time to first flatus crossed back to the futility area since the 6th study and entered the significance zone in favor of the IVF-Lido group since the 8th study (Figure 4A). Besides, both Z curves crossed the calculated line of the required information size since the 7th and 5th studies, respectively. Based on the accumulation of pooled estimates through sequential analyses, the significant benefits of IVF-lido on both endpoints may be true positive results with adequate cases when enrolment of all included studies. Nevertheless, diversities were still high with D^2^ values of 91 and 85% in time to first flatus and defecation, respectively. We further determined the strength of evidence in RCTs only with low or some-concerned overall RoB by using TSA. The Z curves in the study endpoints of time to first flatus (Supplementary Figure 3A) and time to first defecation (Supplementary Figure 3B) neither straddled the O'Brien–Fleming α-spending monitoring boundaries nor reached the futility area. Moreover, the cumulative z curve did not cross the line of the required information size. Therefore, the benefits of IVF-Lido could be inconclusive in RCTs with low or some-concerned overall RoB.

### GRADE Assessment

**Figure 5:**
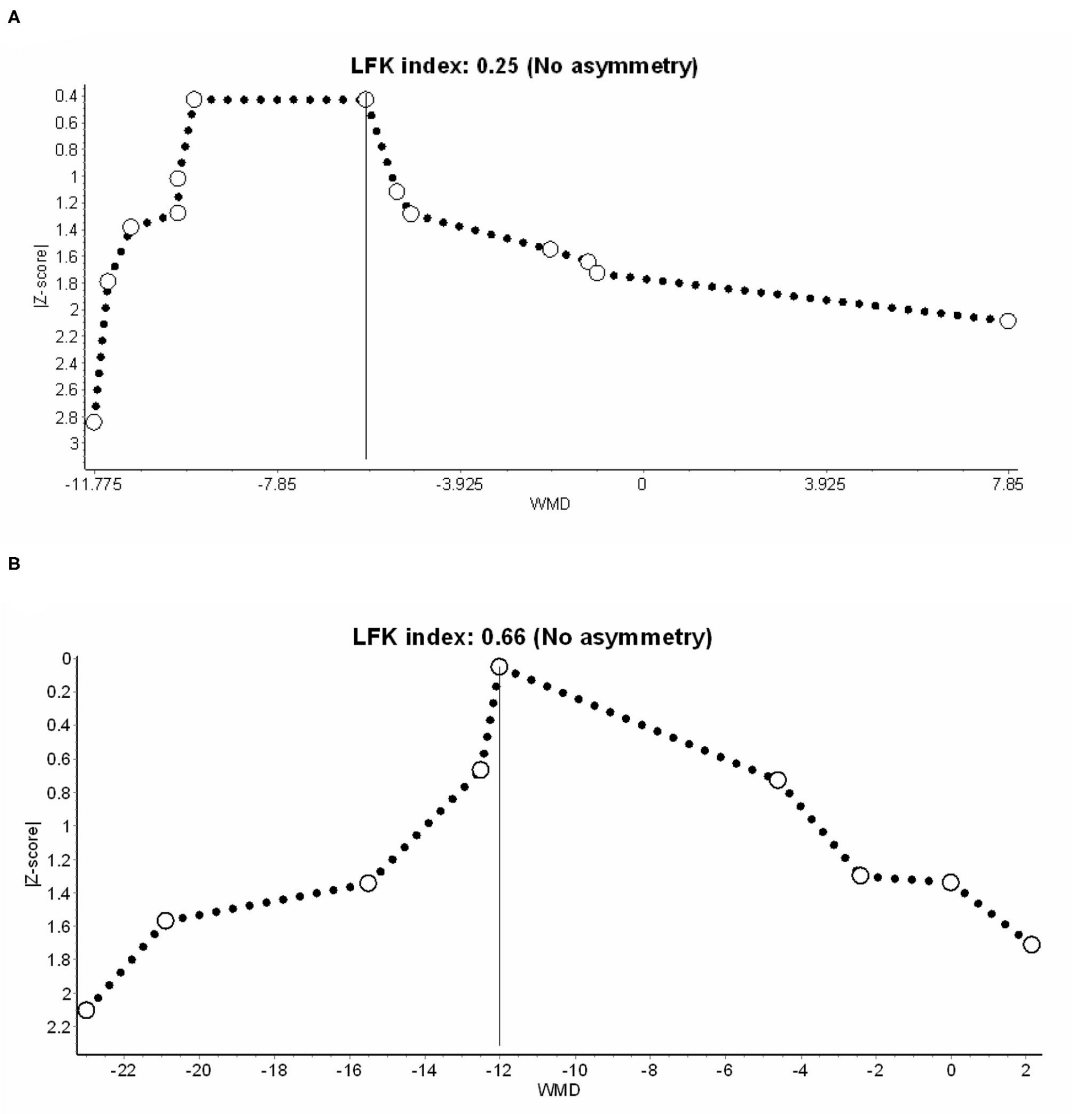
Publication bias in **(A)** time to first flatus and **(B)** defecation, presented with the Doi plot. LFK index, Luis Furuya–Kanamori index.

Table 2 summarizes the CoE of outcomes. Considering that more than half of the enrolled RCTs were rated as some-concerned or high overall RoB, we downgraded the CoE in the domain of RoB. The domain of inconsistency was similarly downgraded because of high heterogeneity in both outcomes. We did not downgrade in the domain of imprecision, because the sample sizes in TSA are sufficient, and the ranges of confidence intervals are acceptable. Publication bias was not considered based on no asymmetry in Doi plots (Figure 5). Finally, the CoE of the benefits from IVF-Lido for both time to first flatus and time to the first defecation was low.

**Table 2:** GRADE assessment.

**Certainty assessment**							**Risk difference with IVF of lidocaine**
**Participants (studies) Follow up**	**Study limitation**	**Inconsistency**	**Indirectness**	**Imprecision**	**Publication bias**	**Overall certainty of evidence**	
**First flatus**							
696 (13 RCTs)	Serious^a^	Serious^b^	Not serious	Not serious	Undetected	⊕⊕○○ LOW	MD **6.03 lower** (8.80 lower to 3.26 lower)
**First defecation**							
498 (9 RCTs)	Serious^a^	Serious^b^	Not serious	Not serious	Undetected	⊕⊕○○ LOW	MD **10.49 lower** (15.58 lower to 5.41 lower)

*IVF, intravenous infusion; RCT, randomized controlled trial; RoB, risk of bias; CI: confidence interval; MD, mean difference; GRADE, grading of recommendations, assessment, development, and evaluations*.

a *≥ 50% enrolled RCTs were with some-concerned or high overall RoB*.

b *I^2^ > 60%*.

### The Role of Analgesic Effects From IVF-Lido in Meta-Regression

**Figure 6:**
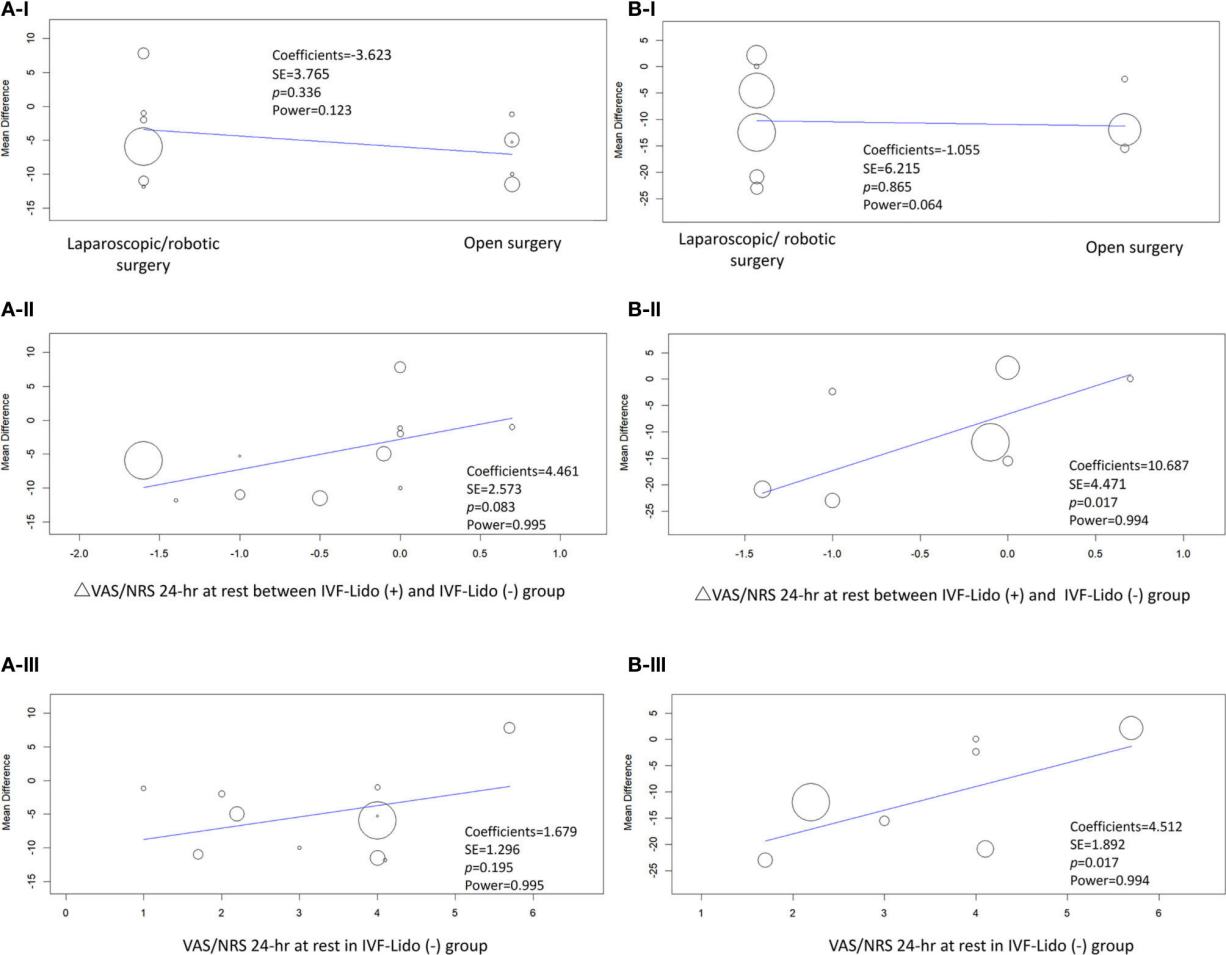
Meta-regression of studies in **(A)** time to first flatus and **(B)** defecation. Covariates included **(I)** open or laparoscopic/robotic surgery, **(II)** changes (Δ) in the pain score for 24 h at rest between IVF-Lido (+) and IVF-Lido (-) groups; **(III)** the pain score for 24 h at rest in the IVF-Lido (-) group was analyzed. IVF-Lido, intravenous infusion of lidocaine; VAS, visual analog scale; NRS, numeric rating scale; SE, standard error; h, hour.

Considering high heterogeneity of the pooled effects of RCTs, we performed sensitivity analyses using meta-regression. Firstly, considering that the different types of surgeries (laparoscopic/robotic and open) might lead to different wound sizes and influence the analgesic effects of IVF-Lido, we analyzed the data by using “surgical type” as a covariant. The results showed that the mean differences in time to first flatus (Figure 6A-I) and defecation (Figure 6B-I) were not remarkably influenced by the type of surgeries, but the power was very low in both outcomes (0.123 and.064, respectively). Secondly, considering that the speed of bowel function recovery might be correlated with different statuses of achieved analgesia, changes of VAS/NRS were used as a covariate for meta-regression. Despite the absence of statistical significance, the more pain alleviated by IVF-Lido, the shorter the time to first flatus appeared with sufficient power (Figure 6A-II). Similarly, a significant correlation between pain relief and time to the first defecation by IVF-Lido was observed (Figure 6B-II), suggesting a potential benefit of IVF-Lido to achieve analgesia. Thirdly, postoperative VAS/NRS at 24 h in the control group was chosen as a covariate to represent the baseline condition of pain control. When more satisfactory analgesia in the control group was achieved, although no significance was observed, a shorter time to first flatus in the IVF-Lido group was depicted (Figure 6A-III). Moreover, the better analgesia in the control group, the significantly shorter time to the first defecation by IVF-Lido was presented (Figure 6B-III).

## Discussion

In comparison with the previous three meta-analyses investigating the effects of IVF-Lido for bowel function recovery after major colorectal surgery in recent 2 years, our updated meta-analysis focused on a high-quality systematic review appraised by AMSTAR2 (Supplementary Table 2) (17–19). Besides, we crossed the language barrier to include three more studies in Chinese and enrolled updated RCTs, and the results in bowel function recovery demonstrated significant enhancement in the administration of intravenous lidocaine in both Asian and non-Asian studies. This study was more comprehensive than the previous meta-analyses.

While following the appraisal criteria described by Chalmers and Jadad (17) or the old Cochrane RoB tool (18, 19), all the previous three meta-analyses did not focus on the potential influence of RCTs by high RoB in the pooled analysis (Supplementary Table 2). RoB 2.0 is, currently, the standard tool recommended by Cochrane Reviews (34), and it is structured into more attentive aspects of domains of bias for an overall quality rating of an RCT. This process provides a more comprehensive and objective quality appraisal, as indicated in our updated meta-analysis when high-overall-RoB studies were excluded. Furthermore, we applied new methodologies, such as TSA and the Doi plot, as assistant tools for advanced and rigorous rating of CoE in GRADE. Castellini et al. (35) reported that TSA adoption would lead to more frequent downgrading of the CoE and could be a supplement for an improved assessment of imprecision in GRADE. We preferred using the Doi plot because the LFK index of the Doi plot demonstrated higher sensitivity to judge publication bias than Egger's regression test (32). Based on the low CoE defined by the GRADE Working Group, the estimation of the pooled effect in meta-analysis might be different from the absolute true effect (36). We tried to analyze the pooled effects from RCTs with low and some-concerned overall RoB, and the benefits of lidocaine disappeared. Insufficient samples with inconclusive results were observed in TSA, and the CoE became very low (Supplementary Table 3). Therefore, more rigorous RCTs on this topic are needed. According to current pieces of evidence, we discretionarily suggest the use of IVF-Lido to facilitate bowel function recovery for major colorectal surgery.

After major colorectal surgery, reducing the pain triggered by a combination of neural and inflammatory pathways facilitates bowel functional recovery (3). Although lidocaine is an amide local anesthetic agent, it has potential benefits for pain relief when applied by perioperative intravenous administration (37). However, discordant findings in previous meta-analyses have raised questions about the relationship between IVF-Lido and postoperative pain control in patients undergoing major colorectal surgery (17–19). The difference of reduction in postoperative pain scores, even if significant, did not meet the threshold of clinically relevant difference of 1 cm in VAS (18). Besides, very high heterogeneity of changes in the pain score and the opioid requirement was observed in the previous meta-analysis, causing uncertainty about the analgesic effect of IVF-Lido. This finding can be explained by the fact that the analgesic property of lidocaine might be insufficient in such low plasma concentrations, and this condition would only block a very small proportion of neuronal sodium channels in these IVF-Lido studies (37). Reducing the use of opioids is considered beneficial to the recovery of bowel function; hence, opioid-sparing analgesic properties have also been discussed in a previous meta-analysis (18). In comparison with the control group, postoperative requirement of morphine requirement did not reach statistical significance in the IVD-Lido group in open or laparoscopic surgeries. We tried to determine the difference in overall morphine consumption between the two groups and found that variant opioids (e.g., morphine, fentanyl, sufentanil, and piritramide) had varying collection times (24–96 h) in the enrolled studies. Therefore, the mechanism in which IVF-Lido can provide enough pain reduction through its analgesic and opioid-sparing mechanism and to the possibility of subsequent rapid bowel function recovery are difficult to explain.

Despite these controversies, the results from our meta-regression demonstrated a positive correlation of faster bowel function recovery associated with more VAS/NRS improvement by IVF-Lido, especially in time to the first defecation. Traditionally, meta-regression is conducted when a meta-analysis under consideration of power has more than 10 studies, but this rule is not strict (30, 38). We calculated the power of meta-analysis, and the power is sufficient in both outcomes to discuss the covariates of analgesic issues, even though only nine studies in the endpoint of time to first defecation. Theoretically, the different agents for pain control in the experimental and control groups should be only IVF-Lido. However, additional analgesics by several regimens were prescribed differently in these studies and may influence the VAS/NRS in the IVF-Lido group. Very high variants in hours of first flatus and defecation among the enrolled studies may be affected by many factors, such as an operator's skills, types of surgeries, and underlying conditions of the patients. All abovementioned factors may affect the relationship between the analgesic effects and bowel function recovery. Besides, the determination of the dose-dependent effect of IVF-Lido on bowel function recovery by meta-regression analysis is limited by the absence of unified dosage and duration in protocols of all included RCTs and the difficulty in the calculation of dosage additional analgesics per hour per kg is the major limitation. Regardless of these limitations, the evidence from our study suggested that the analgesic effects from IVF-Lido still exist.

Surprisingly, in the enrolled RCTs, the beneficial effects persisted for many hours or even days after infusion, which raised a question on the pure analgesic effect of IVF-Lido on bowel function recovery (37, 39). The result from meta-regression with faster bowel function recovery appeared in the IVF-Lido group, while postoperative pain was already under control by other analgesic agents (i.e., best practice in pain control). The mechanism of action through which IVF-Lido can achieve this effect remains unknown. Presumably, anti-inflammation can be a mechanism of IVF-Lido on bowel function recovery based on preclinical investigations (37, 39). Lidocaine, which does not work through sodium channel blockage, reportedly inhibits leukocyte activation and adhesion to injured sites (37; 39). Moreover, lidocaine protects cells from inflammation by inhibiting cytokine production and oxidative stress. The study by Herroeder et al. (11), one of the RCTs with low RoB enrolled in our meta-analysis, echoed such a mechanism. The changes of transcriptional and translational cytokines influence bowel function after a certain period. Hence, the effect was more obvious and significant in the outcome of hours to first defecation (mean: 52.1; 95% CI: 39.7–64.4) compared with hours to first flatus (mean: 38.3; 95% CI: 29.4–47.3) in the IVF-Lido group. This hypothesis can be confirmed by employing more RCTs with specific protocols to confirm the anti-inflammatory mechanism of IVF-Lido on bowel function recovery.

In short, the results from our meta-regression indicate that IVF-Lido might play a multifaceted role in bowel function recovery, including analgesic effect, but other mechanisms (e.g., anti-inflammatory effect) should also be considered. Theoretically, using IVF-Lido in a higher dose and a longer duration fashion might augment the shortening of bowel function recovery time through anti-inflammation and improved analgesic effects. However, physicians should always consider the adverse effects (AEs) and toxicity. Regarding the occurrence of AEs during IVF-Lido, only two of the included RCTs were mentioned in this study (13, 15). No AE or no reports of AEs were observed in the other eleven included RCTs. Therefore, there were insufficient data to subsequentially analyze the safety issue of IVF-Lido. In these two RCTs, the reports of AE included transient confusion, bradycardia without the need for atropine use, and nausea and/or vomiting. Perioperative use of IVF-Lido for postoperative pain and recovery in a Cochrane library of systematic review demonstrated the similar conclusion that the AEs of IVF-Lido are uncertain with very low CoE because only a small number of studies analyzed the occurrence of AEs (5). The RCT conducted by Herroeder et al. (11) reported the plasma concentration of lidocaine below the levels of toxicities with a zero adverse event, suggesting that there still exists a potential safety margin for higher IVF-Lido dosage. Higher perioperative dosage (i.e., up to 5 mg/kg/h) and longer duration (i.e., longer than 24 h) protocols might offer the potential for more apparently enhanced bowel function recovery in future studies (5).

## Conclusions

In this updated meta-analysis with an enrolment of 13 RCTs, perioperative IVF-Lido significantly decreased the time to first flatus and first defecation after major colorectal surgery. Based on low CoE rated by GRADE methodology, we discretionarily suggest the perioperative use of IVF-Lido in patients undergoing major colorectal surgery for improved postoperative bowel function recovery. Analgesic effects of lidocaine may contribute to the recovery of bowel function from the observation of meta-regression. Additional administration of IVF-Lido in patients with the well-controlled analgesia by other strategies remarkably enhanced the speed of intestinal motility after major colorectal surgery, as depicted in meta-regression. Considering the high heterogeneity, more high-quality RCTs should be conducted to provide complete information for the ERAS society of major colorectal surgery.

## Data Availability Statement

The original contributions presented in the study are included in the article/Supplementary Material, further inquiries can be directed to the corresponding authors.

## Author Contributions

P-CC: conceptualization and drafting. PL and YH: methodology, software, formal analysis, and visualization. C-JF, C-HL, and P-CC: validation. P-CC and C-JF: searching and data extraction. C-HL: writing-review and editing. All authors have read and agreed to the published version of the manuscript.

## Conflict of Interest

The authors declare that the research was conducted in the absence of any commercial or financial relationships that could be construed as a potential conflict of interest.

## Publisher's Note

All claims expressed in this article are solely those of the authors and do not necessarily represent those of their affiliated organizations, or those of the publisher, the editors and the reviewers. Any product that may be evaluated in this article, or claim that may be made by its manufacturer, is not guaranteed or endorsed by the publisher.
