# Supplementary material for: Intravenous Infusion of Lidocaine for Bowel Function Recovery After Major Colorectal Surgery: A Critical Appraisal Through Updated Meta-Analysis, Trial Sequential Analysis, Certainty of Evidence, and Meta-Regression
Source: Front Med (Lausanne). 2022 Jan 27;8:759215. doi: 10.3389/fmed.2021.759215 (PMC8828648; doi:10.3389/fmed.2021.759215)
Supplement: Supplementary file 1 [file Data_Sheet_1.pdf]

Supplementary Material

1 Supplementary Figures and Tables

1.1 Supplementary Figures

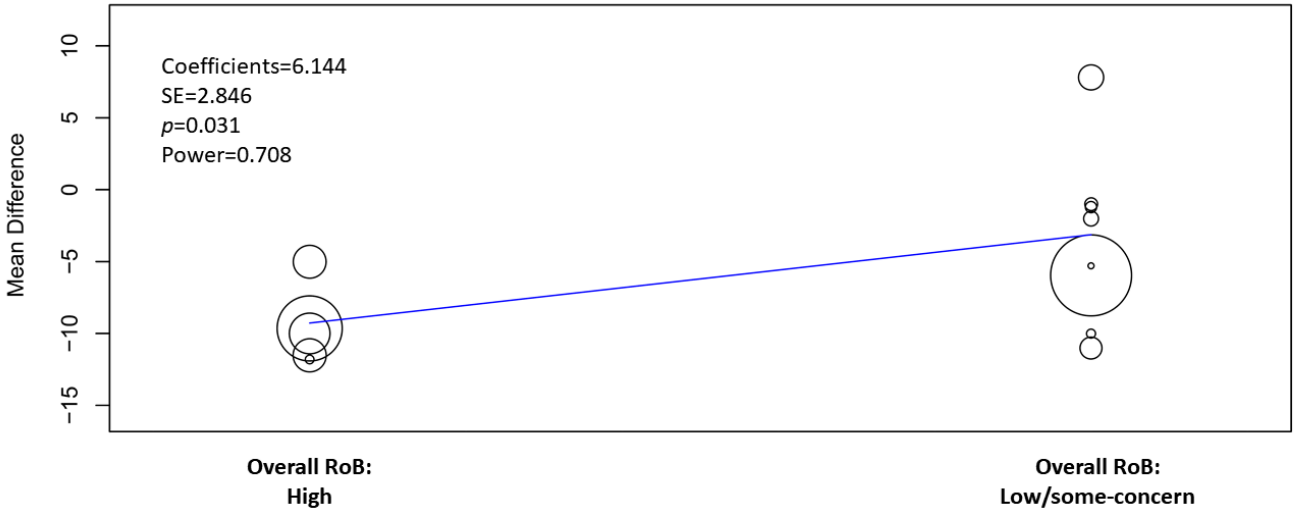

Supplementary Figure 1. Meta-regression between two subgroups, high and low/some-concern overall RoB, in the outcome of time to first flatus. RoB: risk of bias; SE: standard error.

(A)

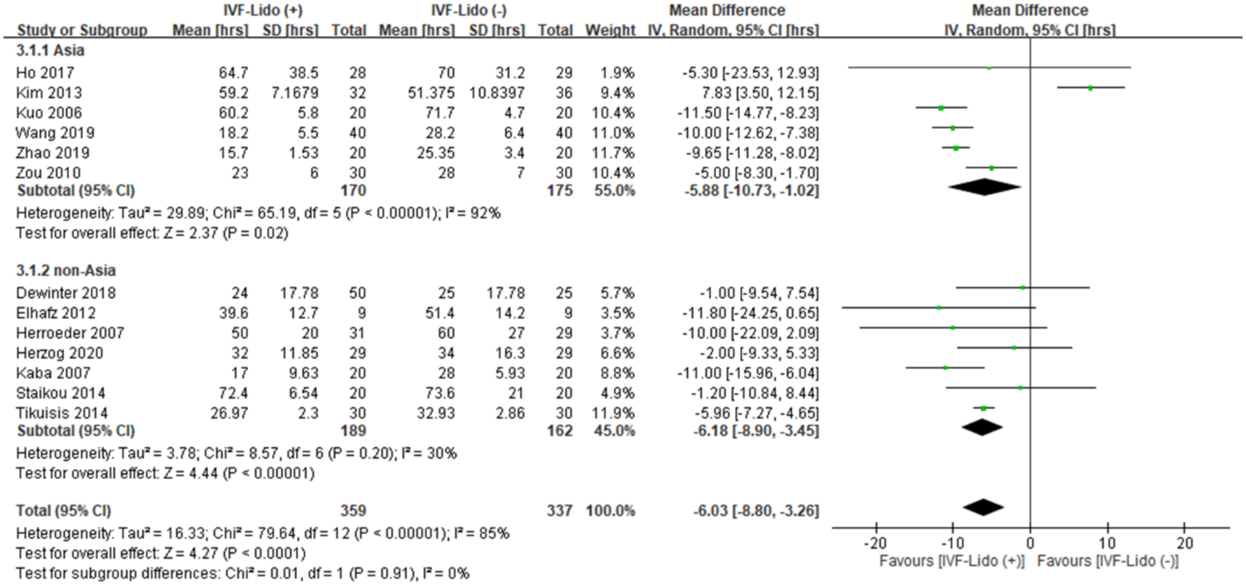

(B)

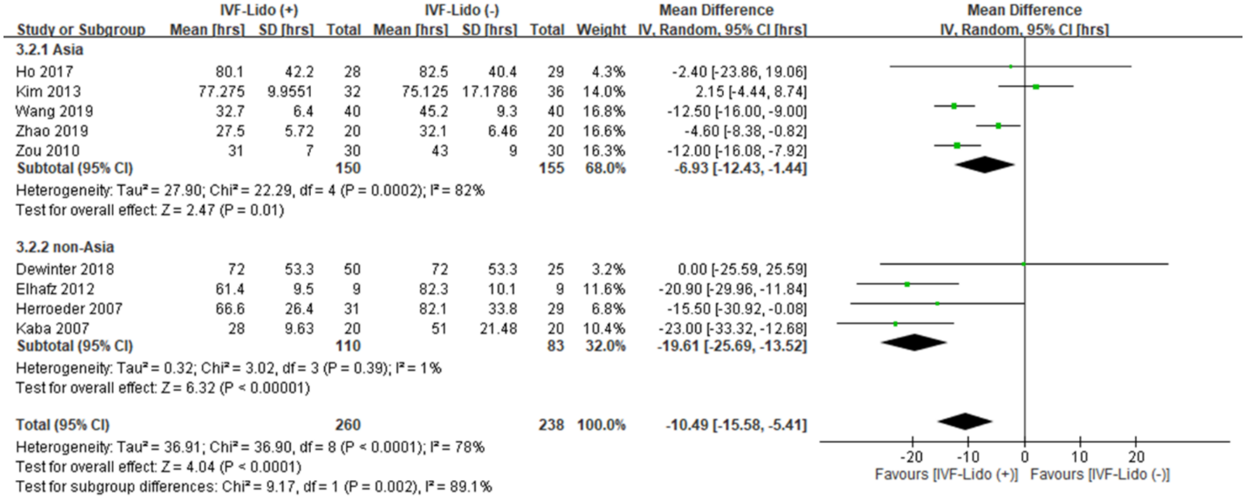

Supplementary Figure 2. Effect of intravenous infusion of lidocaine on the time (hours) to (A) first flatus and (B) defecation. Forest plot with subgroup analysis divided into two groups based on population of the included studies, namely, Asia and non-Asia. IVF-Lido: intravenous infusion of lidocaine; SD: standard deviation; CI: confidence interval.

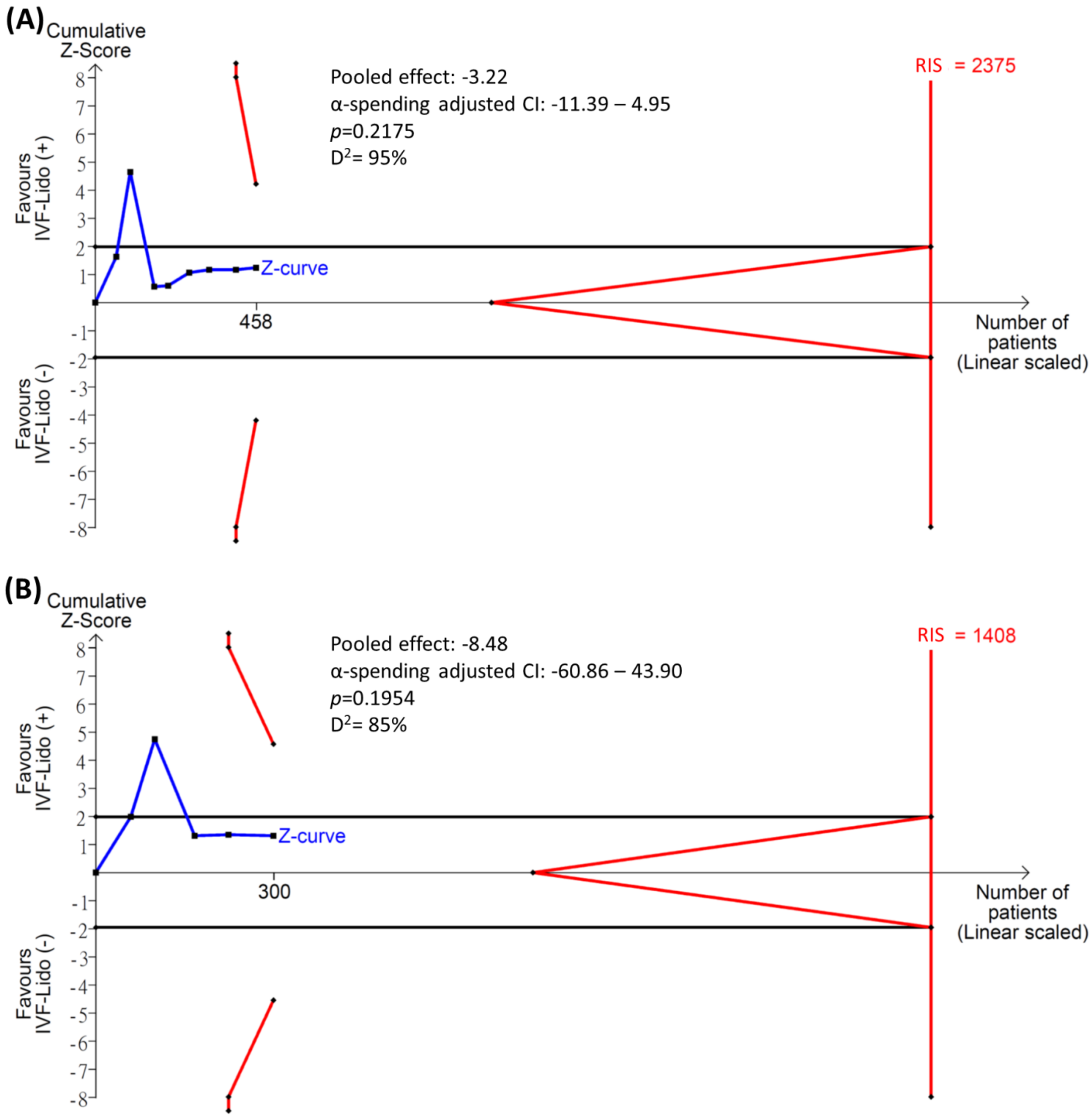

Supplementary Figure 3. Trial sequential analysis of RCTs with low and some-concern overall risk of bias in the time (hours) to (A) first flatus and (B) first defecation**.** IVF-Lido: intravenous infusion of lidocaine; RIS: required information size.

1.2 Supplementary Tables

Supplemental Table1. Search Strategy and results

Supplemental Table 2. Appraisal of currently available systematic review by AMSTAR2

Supplemental Table 3. Certainty of evidence based on RCTs with low and some-concern overall RoB appraised by GRADE
