# Supplementary material for: Intravenous Infusion of Lidocaine for Bowel Function Recovery After Major Colorectal Surgery: A Critical Appraisal Through Updated Meta-Analysis, Trial Sequential Analysis, Certainty of Evidence, and Meta-Regression
Source: Front Med (Lausanne). 2022 Jan 27;8:759215. doi: 10.3389/fmed.2021.759215 (PMC8828648; doi:10.3389/fmed.2021.759215)
Supplement: Supplementary file 2 [file Table_1.DOCX]

**Supplemental Table1.** Search Strategy and results

| **Database** | **#** | **Search syntax** | **Results** |
| --- | --- | --- | --- |
| **Embase** | 1 | (**(**(colon* OR colorectal OR colic OR rectal OR rectum* OR sigmoid OR appendix* OR cecum OR ceca* OR caecum OR caeca* OR anus* OR "anal canal" OR hemorrhoid* OR haemorrhoid* OR pile* OR "blind gut" OR "large bowel*" OR "large intestin*" OR abdominal) NEAR/4 (surg* OR resect* OR operat* OR excis* OR bypass* OR transplant* OR anastomos* OR intraoperat* OR perioperat* OR postoperat* OR intrasurg* OR perisurg* OR postsurg*)**)** **OR** colectom* OR colostom* OR rectostom* OR proctostom* OR appendectom* OR ileocecostom*):ti,ab,kw,de |  |
|  | 2 | "large intestine disease"/exp/dm_su OR "large intestine tumor"/exp/dm_su OR "inflammatory bowel disease"/exp/dm_su **OR** (("large intestine disease"/exp OR "large intestine tumor"/exp OR "inflammatory bowel disease"/exp) AND ("postoperative complication"/exp OR "postoperative pain"/exp OR "postoperative period"/exp OR "peroperative complication"/exp OR "perioperative period"/exp OR "intraoperative period"/exp)) **OR** "intestine surgery"/de OR "appendectomy"/exp OR "colon surgery"/exp OR "colorectal surgery"/exp OR "intestine anastomosis"/exp OR "intestine bypass"/exp OR "intestine resection"/exp OR "intestine transplantation"/exp OR "rectum surgery"/exp |  |
|  | 3 | (lidocain* OR lignocain* OR xylocain*):ti,ab,kw,de |  |
|  | 4 | [lidocaine](https://www.ncbi.nlm.nih.gov/pubmed/30721376)/exp |  |
|  | 5 | (#1 OR #2) AND (#3 OR #4) AND [embase]/lim |  |
|  | 6 | #6 AND ('crossover procedure':de OR 'double-blind procedure':de OR 'randomized controlled trial':de OR 'single-blind procedure':de OR (random* OR  factorial* OR crossover* OR cross NEXT/1 over* OR placebo* OR doubl* NEAR/1 blind* OR singl* NEAR/1 blind* OR assign* OR allocat* OR volunteer*):de,ab,ti) | **RCT: 566** |
| **MEDLINE**  **Ovid** | 1 | (**(**(colon* OR colorectal OR colic OR rectal OR rectum* OR sigmoid OR appendix* OR cecum OR ceca* OR caecum OR caeca* OR anus* OR "anal canal" OR hemorrhoid* OR haemorrhoid* OR pile* OR "blind gut" OR "large bowel*" OR "large intestin*" OR abdom*) ADJ4 (surg* OR resect* OR operat* OR excis* OR bypass* OR transplant* OR anastomos* OR intraoperat* OR perioperat* OR postoperat* OR intrasurg* OR perisurg* OR postsurg*)**)** **OR** colectom* OR colostom* OR rectostom* OR proctostom* OR appendectom* OR ileocecostom*).mp |  |
|  | 2 | ((exp "Cecal Diseases"/ OR exp "Colonic Diseases"/ OR exp "Rectal Diseases"/ OR exp "Cecal Neoplasms"/ OR exp "Colorectal Neoplasms"/ OR exp "Inflammatory Bowel Diseases"/ OR exp "Intestine, Large"/ OR "Abdomen"/) AND (surgery.fx. OR exp "Perioperative Period"/ OR exp "Perioperative Care"/ OR exp "Postoperative Complications"/)) **OR** exp "Colorectal Surgery"/ OR exp "Appendectomy"/ OR exp "Colectomy"/ OR exp "Cecostomy"/ OR exp "Colostomy"/ OR exp "Proctectomy"/ |  |
|  | 3 | (lidocain* OR lignocain* OR xylocain*).mp |  |
|  | 4 | exp "[Lidocaine](https://www.ncbi.nlm.nih.gov/pubmed/30721376)"/ |  |
|  | 5 | (1 OR 2) AND (3 OR 4) |  |
|  | 6 | 5 AND (randomized controlled trial.pt. or controlled clinical trial.pt. or randomi*ed.ab. or placebo.ab. or drug therapy.fs. or randomly.ab. or trial.ab. or groups.ab. not (exp animals/ not humans.sh.)) | **RCT: 311** |
| **Cochrane Trials** | 1 | (**(**(colon* OR colorectal OR colic OR rectal OR rectum* OR sigmoid OR appendix* OR cecum OR ceca* OR caecum OR caeca* OR anus* OR "anal canal" OR hemorrhoid* OR haemorrhoid* OR pile* OR "blind gut" OR "large bowel*" OR "large intestin*" OR abdominal) NEAR/3 (surg* OR resect* OR operat* OR excis* OR bypass* OR transplant* OR anastomos* OR intraoperat* OR perioperat* OR postoperat* OR intrasurg* OR perisurg* OR postsurg*)**)** **OR** colectom* OR colostom* OR rectostom* OR proctostom* OR appendectom* OR ileocecostom*):ti,ab,kw |  |
|  | 2 | (([mh "Cecal Diseases"] OR [mh "Colonic Diseases"] OR [mh "Rectal Diseases"] OR [mh "Cecal Neoplasms"] OR [mh "Colorectal Neoplasms"] OR [mh "Inflammatory Bowel Diseases"] OR [mh "Intestine, Large"] OR [mh ^"Abdomen"]) AND ([mh /SU] OR [mh "Perioperative Period"] OR [mh "Perioperative Care"] OR [mh "Postoperative Complications"])) **OR** [mh "Colorectal Surgery"] OR [mh "Appendectomy"] OR [mh "Colectomy"] OR [mh "Cecostomy"] OR [mh "Colostomy"] OR [mh "Proctectomy"] |  |
|  | 3 | (lidocain* OR lignocain* OR xylocain*):ti,ab,kw |  |
|  | 4 | [mh "[Lidocaine](https://www.ncbi.nlm.nih.gov/pubmed/30721376)"] |  |
|  | 5 | (#1 OR #2) AND (#3 OR #4) |  |
|  | 6 | #5 in Trials  non-registered: 310  register from CT.gov and ICTRP: 167 | **RCT: 477** |
| **Scopus** | 1 | TITLE-ABS-KEY (**(**(colon* OR colorectal OR colic OR rectal OR rectum* OR sigmoid OR appendix* OR cecum OR ceca* OR caecum OR caeca* OR anus* OR "anal canal" OR hemorrhoid* OR haemorrhoid* OR pile* OR "blind gut" OR "large bowel*" OR "large intestin*" OR abdom*) W/3 (surg* OR resect* OR operat* OR excis* OR bypass* OR transplant* OR anastomos* OR intraoperat* OR perioperat* OR postoperat* OR intrasurg* OR perisurg* OR postsurg*)**)** **OR** colectom* OR colostom* OR rectostom* OR proctostom* OR appendectom* OR ileocecostom*) |  |
|  | 2 | TITLE-ABS-KEY (lidocain* OR lignocain* OR xylocain*) |  |
|  | 3 | TITLE-ABS-KEY (random* OR placebo*) OR TITLE (trial) |  |
|  | 4 | #1 AND #2 AND #3 | **RCT: 455** |
| **China National Knowledge Infrastructure (CNKI)** | 1 | SU="大腸" OR SU="直腸" OR SU="結腸" OR SU="盲腸" OR SU="肛門" OR SU="腹部" OR SU="colon" OR SU="colorectal" OR SU="colic" OR SU="rectal" OR SU="rectum" OR SU="sigmoid" OR SU="appendix" OR SU="cecum" OR SU="ceca" OR SU="cecal" OR SU="caecum" OR SU="caeca" OR SU="caecal" OR SU="anus" OR SU="anuses" OR SU=""anal canal"" OR SU="hemorrhoid" OR SU="hemorrhoids" OR SU="haemorrhoid" OR SU="haemorrhoids" OR SU="pile*" OR SU="blind gut" OR SU="large bowel" OR SU="large bowels" OR SU="large intestine" OR SU="large intestines" OR SU="large intestinal" OR SU="abdomen" OR SU="abdomens" OR SU="abdominal" |  |
|  | 2 | SU="手術" OR SU="外科" OR SU="開刀" OR SU="切除" OR SU="摘除" OR SU="繞道" OR SU="移植" OR SU="吻合" OR SU="術後" OR SU="術期" OR SU="腸術" OR surgery" OR SU="surgical" OR SU="surgical" OR SU="resection" OR SU="resections" OR SU="operation" OR SU="operations" OR SU="operative" OR SU="operatively" OR SU="excision" OR SU="excisions" OR SU="bypass" OR SU="bypasses" OR SU="transplant" OR SU="transplantation" OR SU="transplantations" OR SU="anastomose" OR SU="anastomoses" OR SU="intraoperative" OR SU="perioperative" OR SU="postoperative" OR SU="intrasurgery" OR SU="perisurgery" OR SU="postsurgery" OR SU="intrasurgerical" OR SU="perisurgerical" OR SU="postsurgerical" |  |
|  | 3 | SU="利多卡因" OR SU="利度卡因" OR SU="苦息樂卡因 OR SU="lidocaine" OR SU="lignocaine" OR SU="xylocaine" OR SU="lidocaines" OR SU="lignocaines" OR SU="xylocaines" |  |
|  | 4 | #1 AND #2 AND #3 | **All: 458** |
| **Index to Taiwan Periodical Literature System** |  | (大腸 + 直腸 + 結腸 + 盲腸 + 肛門 + 腹部 + colon + colorectal + colic + rectal + rectum + sigmoid + appendix + cecum + ceca + cecal + caecum + caeca + caecal + anus + anuses + anal canal + hemorrhoid + hemorrhoids + haemorrhoid + haemorrhoids + pile* + blind gut + large bowel + large bowels + large intestine + large intestines + large intestinal + abdomen + abdomens + abdominal) * (手術 + 外科 + 開刀 + 切除 + 摘除 + 繞道 + 移植 + 吻合 + 術後 + 術期 + 腸術 + surgery + surgical + surgical + resection + resections + operation + operations + operative + operatively + excision + excisions + bypass + bypasses + transplant + transplantation + transplantations + anastomose + anastomoses + intraoperative + perioperative + postoperative + intrasurgery + perisurgery + postsurgery + intrasurgerical + perisurgerical + postsurgerical) * (利多卡因 + 利度卡因 + 苦息樂卡因 + lidocaine + lignocaine + xylocaine + lidocaines + lignocaines + xylocaines) | **All: 5** |
| **ICTRP** |  | **Condition:** **(**(colo* OR colic OR rectal OR rectu* OR sigm* OR appen* OR cecum OR ceca* OR "large bo*" OR "large intes*" OR abdo*) AND (surg* OR resec* OR oper* OR excis* OR bypass* OR transp* OR anast* OR intraop* OR periop* OR postop* OR intrasu* OR perisu* OR postsu*)**)**  **AND**  **Intervention:** (lidocain* OR lignocain* OR xylocain*) | **RCT: 26** |
