# Supplementary material for: Intravenous Infusion of Lidocaine for Bowel Function Recovery After Major Colorectal Surgery: A Critical Appraisal Through Updated Meta-Analysis, Trial Sequential Analysis, Certainty of Evidence, and Meta-Regression
Source: Front Med (Lausanne). 2022 Jan 27;8:759215. doi: 10.3389/fmed.2021.759215 (PMC8828648; doi:10.3389/fmed.2021.759215)
Supplement: Supplementary file 3 [file Table_2.DOCX]

**Supplemental Table 2.** Appraisal of currently available systematic review by AMSTAR2.

| **No.** | **Items** | **References** | | | |
| --- | --- | --- | --- | --- | --- |
|  |  | Cooke *el al.*^17^ (2019) | Wei *et al.* ^18^ (2020) | Rollins *et al.* ^19^ (2020) | Present study  (2021) |
| *1.* | *Coincident PICO in this S/R.* | *Yes* | *Yes* | *Yes* | *Yes* |
| *2.* | ***Protocolized processing of S/R****.* | *Partial Yes* | *Partial Yes* | *Partial Yes* | *Yes* |
| *3.* | *Explain the selection of study type.* | ***No*** | ***No*** | ***No*** | *Yes* |
| *4.* | ***Comprehensive search strategy.*** | ***No*** | *Partial Yes* | *Yes* | *Yes* |
| *5.* | *Double check the selected studies.* | ***No*** | ***No*** | *Yes* | *Yes* |
| *6.* | *Double check data extraction.* | *Yes* | ***No*** | *Yes* | *Yes* |
| *7.* | ***List and explanation of excluded studies.*** | *Yes* | *Yes* | *Yes* | *Yes* |
| *8.* | *Describe the details of included studies.* | *Yes* | *Yes* | *Yes* | *Yes* |
| *9.* | ***Satisfactory technique for assessing the RoB.*** | *Yes* | *Yes* | *Yes* | *Yes* |
| *10.* | *Report on the sources of funding in the enrolled studies.* | ***No*** | ***No*** | ***No*** | *Yes* |
| *11.* | ***Appropriate methods for statistical combination.*** | *Yes* | *Yes* | *Yes* | *Yes* |
| *12.* | *Assess the potential impact of RoB in studies on the M/A results* | ***No*** | ***No*** | ***No*** | *Yes* |
| *13.* | ***Discuss the influence of RoB on the M/A results.*** | ***No*** | ***No*** | ***No*** | *Yes* |
| *14.* | *Explain or discuss the heterogeneity.* | ***No*** | *Yes* | *Yes* | *Yes* |
| *15.* | ***Analyze and discuss publication bias.*** | ***No*** | ***No*** | ***No*** | *Yes* |
| *16.* | *Report COI to conduct this S/R M/A.* | *Yes* | *Yes* | *Yes* | *Yes* |
| **Overall score** | | ***Critically low*** | ***Critically low*** | ***Critically low*** | ***High*** |

1. Abbreviations: AMSTAR = A MeaSurement Tool to Assess systematic Reviews; PICO = population, intervention, comparison, and outcome; S/R = systemic review; M/A = meta-analysis; RoB = risk of bias; COI = conflict of interest.
2. Boldface of the words in the domains indicates the critical domain of AMSTAR2. Critically low is rated when a S/R has more than one flaw in critical domain, which may not provide an accurate and comprehensive summary in this topic.
