# Supplementary material for: Intravenous Infusion of Lidocaine for Bowel Function Recovery After Major Colorectal Surgery: A Critical Appraisal Through Updated Meta-Analysis, Trial Sequential Analysis, Certainty of Evidence, and Meta-Regression
Source: Front Med (Lausanne). 2022 Jan 27;8:759215. doi: 10.3389/fmed.2021.759215 (PMC8828648; doi:10.3389/fmed.2021.759215)
Supplement: Supplementary file 4 [file Table_3.DOCX]

**Supplemental Table 3:** Certainty of evidence based on RCTs with low and some-concern overall RoB appraised by GRADE

|  | | | | | | | |
| --- | --- | --- | --- | --- | --- | --- | --- |
| **Certainty assessment** | | | | | | | **Risk difference with IVF of lidocaine** |
| **Participants  (studies) Follow up** | **Study limitation** | **Inconsistency** | **Indirectness** | **Imprecision** | **Publication bias** | **Overall certainty of evidence** |  |
| **First flatus –** **exclusion of RCTs with overall high RoB** | | | | | | | |
| 458 (8 RCTs) | serious ^a^ | serious ^b^ | not serious | very serious ^c^ | publication bias strongly suspected ^d^ | ⨁◯◯◯ VERY LOW | MD **3.22 lower** (8.33 lower to 1.9 higher) |
| **First defecation - exclusion of RCTs with overall high RoB** | | | | | | | |
| 300 (5 RCTs) | serious ^a^ | serious ^b^ | not serious | very serious ^c^ | publication bias strongly suspected ^d^ | ⨁◯◯◯ VERY LOW | MD **8.48 lower** (21.31 lower to 4.36 higher) |

**IVF:** intravenous infusion; **RCT:** randomized controlled trial; **RoB:** risk of bias; **CI:** Confidence interval; **MD:** Mean difference

#### Explanations:

a. ≥50% enrolled RCTs were overall RoB with some concerns

b. I^2^ > 60%

c. Did not reach required information size and wide confidence interval

d. Doi plot showed asymmetry
